# Suitable extracellular oxidoreduction potential inhibit *rex* regulation and effect central carbon and energy metabolism in *Saccharopolyspora spinosa*

**DOI:** 10.1186/s12934-014-0098-z

**Published:** 2014-08-27

**Authors:** Xiangmei Zhang, Chaoyou Xue, Fanglong Zhao, Dashuai Li, Jing Yin, Chuanbo Zhang, Qinggele Caiyin, Wenyu Lu

**Affiliations:** Department of Biological Engineering, School of Chemical Engineering and Technology, Tianjin University, Tianjin, 300072 PR China; Key Laboratory of system bioengineering (Tianjin University), Ministry of Education, Tianjin, 300072 PR China; Collaborative Innovation Center of Chemical Science and Engineering (Tianjin), Tianjin, 300072 PR China

**Keywords:** *Saccharopolyspora spinosa*, Oxidative condition, H_2_O_2_, *Rex*, Metabolites

## Abstract

**Background:**

Polyketides, such as spinosad, are mainly synthesized in the stationary phase of the fermentation. The synthesis of these compounds requires many primary metabolites, such as acetyl-CoA, propinyl-CoA, NADPH, and succinyl-CoA. Their synthesis is also significantly influenced by NADH/NAD^+^. *Rex* is the sensor of NADH/NAD^+^ redox state, whose structure is under the control of NADH/NAD^+^ ratio. The structure of *rex* controls the expression of many NADH dehydrogenases genes and cytochrome *bd* genes. Intracellular redox state can be influenced by adding extracellular electron acceptor H_2_O_2_. The effect of extracellular oxidoreduction potential on spinosad production has not been studied. Although extracellular oxidoreduction potential is an important environment effect in polyketides production, it has always been overlooked. Thus, it is important to study the effect of extracellular oxidoreduction potential on *Saccharopolyspora spinosa* growth and spinosad production.

**Results:**

During stationary phase, *S. spinosa* was cultured under oxidative (H_2_O_2_) and reductive (dithiothreitol) conditions. The results show that the yield of spinosad and pseudoaglycone increased 3.11 fold under oxidative condition. As H_2_O_2_ can be served as extracellular electron acceptor, the ratios of NADH/NAD^+^ were measured. We found that the ratio of NADH/NAD^+^ under oxidative condition was much lower than that in the control group. The expression of *cytA* and *cytB* in the *rex* mutant indicated that the expression of these two genes was controlled by *rex*, and it was not activated under oxidative condition. Enzyme activities of PFK, ICDH, and G6PDH and metabolites results indicated that more metabolic flux flow through spinosad synthesis.

**Conclusion:**

The regulation function of rex was inhibited by adding extracellular electron acceptor-H_2_O_2_ in the stationary phase. Under this condition, many NADH dehydrogenases which were used to balance NADH/NAD^+^ by converting useful metabolites to useless metabolites and unefficient terminal oxidases (cytochrome *bd*) were not expressed. So lots of metabolites were not waste to balance. As a result, un-wasted metabolites related to spinosad and PSA synthesis resulted in a high production of spinosad and PSA under oxidative condition.

**Electronic supplementary material:**

The online version of this article (doi:10.1186/s12934-014-0098-z) contains supplementary material, which is available to authorized users.

## Background

Spinosyns containing a 21-carbon tetracyclic lactone are produced by *Saccharopolyspora spinosa* [1]. Besides to the tetracyclic lactone core, spinosyns also contain two deoxysugars, tri-*O*-methylated rhamnose and forosamine. Pseudoaglycones (PSAs) that lack forosamine are direct intermediates of spinosyns. So far, studies have demonstrated that *S. spinosa* can synthesize more than 25 spinosyns that vary in structures and functions [2]. Among these spinosyns, spinosyn A and spinosyn D, the mixture of which was called spinosad, are the most two abundant and effective spinosyns [2]. Spinosad has shown broad-spectrum insecticidal activity and a high level of selectivity and effectivity. More importantly, spinosad has no effect on nontarget insects and mammals [3,4]. Because of these advantages, spinosad-based insect control pesticide was awarded the Presidential Green Chemistry Challenge Award in 1999 [5].

In the last few years, metabolic engineering, classic random mutagenesis, and fermentation process optimization have been used to improve the yield of spinosad [6]. By over-expression rhamnose-synthesizing genes with their own promoter the yield of spinosad was significantly improved [1]. Pan et al. [7] made a three-fold improvement by over-expression rhamnose-synthesizing genes under the control of PermE* promoter. Xue et al. [8] made a five-fold improvement through rational metabolic engineering. For the random mutagenesis, Liang et al. [9] made a 2.86-fold improvement of spinosad though UV mutagenesis. Besides, spinosad production was significantly improved through fermentation media optimization using response surface methodology [10]. However, there is no study on the effect of extracellular oxidoreduction potential (ORP) on *S. spinosa* growth, spinosad production, metabolism changes and enzyme activities.

Spinosad is produced in the stationary phase of the fermentation. Oxygen, however, is not always sufficiently provided in this stage because of the limitation of rotate speeds. The insufficient oxygen in this stage would lead to a rapid increase in the NADH/NAD^+^ ratio. The increase of NADH/NAD^+^ ratio may change DNA binding ability of *rex*, which is a sensor of NADH/NAD^+^ redox state [11]. High NADH/NAD^+^ ratio leads *rex* to lose affinity for target DNA. As a result, inefficient electron transport system-cytochrome *bd* oxidase (*cytABCD*) and many NADH dehydrogenases would be expressed [12]. These NADH dehydrogenases indicate enzymes that contain ‘Rossmann fold’ domain, which is structurally homologous to redox-sensing domain, such as alcohol dehydrogenase and lactate dehydrogenase [12]. The expression of these genes can modulate unbalanced NADH/NAD^+^ ratio at the expense of changing intracellular metabolites to useless byproducts and using inefficient energy producing system (cytochrome *bd* oxidase). The intracellular ORP, which is determined mainly by the ratio of NADH/NAD^+^, can be influenced by changing extracellular ORP. Extracellular ORP can be changed by adding oxidative or reductive substances, such as dithiothreitol (DTT), potassium ferricyanide, dissolved oxygen (DO), and H_2_O_2_ [13,14]. Among these substances, DO and H_2_O_2_ are electron acceptors. Whether and how extracellular ORP change the metabolism of *S. spinosa* would be important, because such information can give us a global metabolic view about the response of *S. spinosa* to the change of extracellular ORP. Besides, many useful clues about how to improve spinosad production can also be obtained.

In this paper, we describe the effect of oxidative condition, created by adding H_2_O_2_ in the stationary phage, on *S. spinosa* growth, spinosad and PSA production, and glucose consumption. Besides, the effect of oxidative condition on NADH/NAD^+^ ratio, gene expression of *cytAB*, activities of key redox-dependent enzymes (PFK, ICDH and G6PDH) in glycolysis, TCA cycle and pentose phosphate pathway (PPP), and intracellular metabolites change were also studied.

## Results

### Spinosad and PSA production and *S. spinosa* growth under different extracellular oxidation-reduction potentials

**Figure 1:**
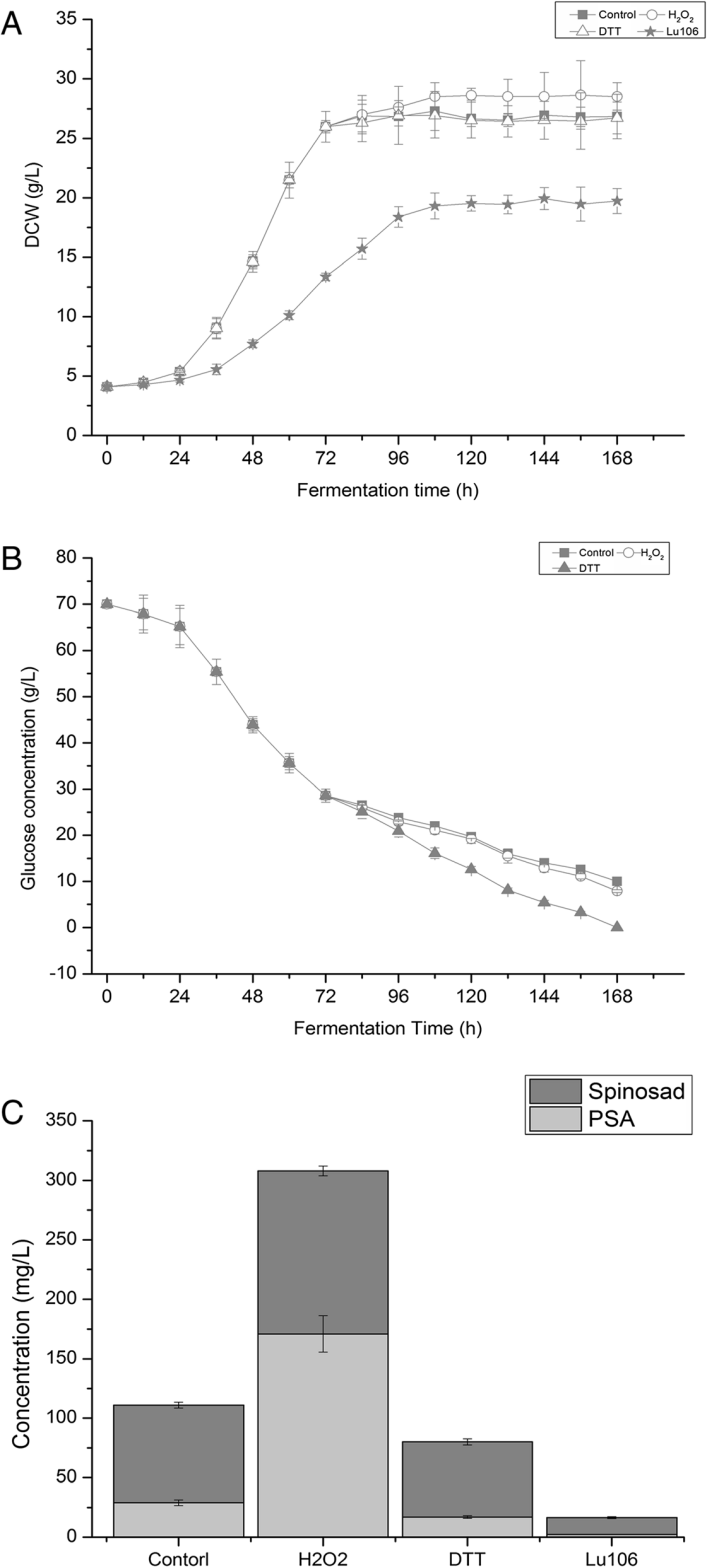
**Effect of different fermentation conditions on cell growth, glucose consumption, spinosad and PSA production of wild-type** ***S. spinosa*** **, and cell growth spinosad and PAS production of** ***rex*** **-mutant Lu106. (A)** Fermentation curve of *rex*-mutant Lu106 under control condition (star) and fermentation curve of wild-type under control condition (square), oxidative condition- H_2_O_2_ (circle), and reductive condition- DTT (triangle); **(B)** Glucose consumption of wild-type under control condition (square), oxidative condition- H_2_O_2_ (circle), and reductive condition- DTT (triangle); **(C)** Spinosad and PSA production of *rex*-mutant Lu106 under control condition and spinosad and PSA production of wild-type under control condition (control), oxidative condition- H_2_O_2_, and reductive condition- DTT.

Batch cell growth, spinosad production, and fermentation parameters were analyzed for the whole fermentation process under different extracellular oxidoreduction potential. Oxidative condition was created by adding 5 mmol/L H_2_O_2_ every 12 h from the initial of stationary stage of the fermentation, 72 h. Reducing condition was created by adding 3 g/L DTT at the initial of stationary stage of the fermentation, 72 h. Because high H_2_O_2_ concentration can jeopardize *S. spinosa* growth, 5 mmol/L H_2_O_2_ was added every 12 h. 5 mmol/L H_2_O_2_ did not affect *S. spinosa* growth and was consumed totally in 12 h (data not shown). Cell growth, spinosad production, and glucose consumption under different conditions were shown in Figure 1. Cell growth between the control group and reducing group shown no difference (Figure 1A). While dry cell weight (DCW) under oxidative condition was increased slightly, less than 4%. In contrast, glucose consumption between control and oxidative condition did not have difference. Glucose consumption rate under reducing condition was increased (Figure 1B). The total yield of spinosad and PSA under oxidative condition reached 308 mg/L, which was 3.11 fold of that in control group (Figure 1C). In contrast, the yield of spinosad and PSA under reducing condition was decreased significantly. Figure 1C shows that oxidative condition in stationary stage of fermentation was favorable for the production of spinosad.

### Intracellular NADH/NAD^+^ levels

**Figure 2:**
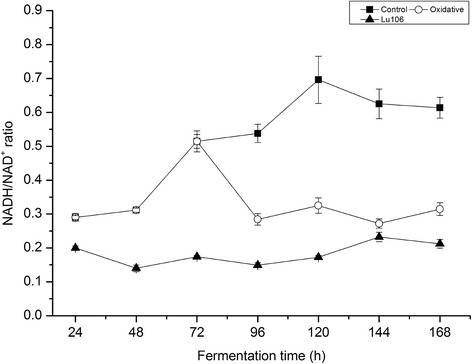
**NADH/NAD** ^**+**^ **ratio of** ***rex*** **-mutant Lu106 under control condition (triangle) and wild-type under control condition (square) and oxidative condition (circle).**

As H_2_O_2_ is an electron acceptor, the differences of the ratios of NADH/NAD^+^ between the control and oxidative condition were analyzed. As shown in Figure 2 the ratios of NADH/NAD^+^ from 24 h to 48 h were maintained about 0.31. Then the ratios of NADH/NAD^+^ were increased and reached 0.52 at 72 h. After 72 h, the ratios of NADH/NAD^+^ in the control group were maintained higher than 0.52, while the ratios of NADH/NAD^+^ under oxidative condition were decreased to and maintained at 0.28 to 0.32. It means that the ratios of NADH/NAD^+^ in the stationary phase were higher than that in the exponential phase in the control group. However, the ratios of NADH/NAD^+^ in the stationary phase were almost the same as that in the exponential phase under oxidative condition (Figure 2). These results indicate that the redox status in *S. spinosa* was significantly influenced.

### *Rex* and cytochrome *bd* oxidase genes determination and expression assays

Studies have demonstrated that the *rex* regulator responds to intracellular NADH/NAD^+^ levels and controls the expression of genes involved in lots of metabolisms in *Actinomycetales* [15]. The complete genome of *S. spinosa* ATCC 49460, accession number NZ_GL877878 in the NCBI nucleotide database (http://www.ncbi.nlm.nih.gov/nuccore/NZ_GL877878.1), was blasted with *rex* in *Saccharopolyspora erythraea*, *Streptomyces coelicolor*, and *Streptomyces avermitilis* by using the BLASTP algorithm with significant sequence similarity (E value < 10^−40^). The *rex* gene in the *S. spinosa* genome sequencing was identified (Additional file 1: Figure S1) [15]. By blasting genes located in the downstream of *rex* with the genome of *Saccharopolyspora erythraea*, *Streptomyces coelicolor*, and *Streptomyces avermitilis*, we found that genes located in the downstream of *rex* were cytochrome *bd* oxidase synthesis gene, *cytAB*.

**Figure 3:**
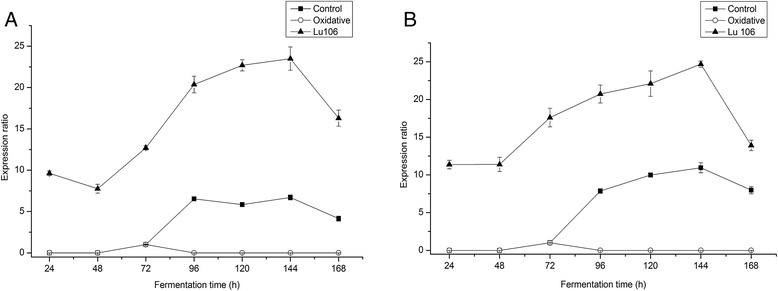
**Gene expression ratios of** ***cytA*** **and** ***cytB*** **.** Relative gene expression ratios of *cytA* **(A)** or *cytB* **(B)** of *rex*-mutant Lu106 under control condition (triangle) and wild-type under control condition (square) and oxidative condition (circle).

The expression of *cytA* and *cytB* were monitored using RT-qPCR to (I) prove that higher NADH/NAD^+^ levels can activate *rex*, the activation of *rex* controls the expression of *cytA* and *cytB*, (II) use the expression of *cytA* and *cytB* to indicate whether *rex* was activated. The expression of *cytA* and *ctyB* in 72 h was assigned as the reference. As shown in Figure 3, *cytA* and *cytB* were not expressed at the lag phase and exponential stage. *cytA* and *cytB* began to express at the initial of stationary phase, 72 h. During the whole stationary phase, *cytA* and *cytB* were expressed continuously in the control group. In contrast, the expression of *cyt A* and *cytB* in the stationary phase was ceased after adding H_2_O_2_ at 72 h (Figure 3). The expression profiles of *ctyA* and *ctyB* both in the control group and the oxidative condition were consistent with NADH/NAD^+^ levels (Figure 2). When the ratio of NADH/NAD^+^ was higher than 0.52 in *S. spinosa*, *rex* had a conformation change and the DNA binding abilities of *rex* were inhibited. As a result, *rex*-regulated genes, such as *cytA* and *cytB*, were expressed (Figure 3).

### *Rex* deletion

To further study the relationship between the expression of *cytAB* and *rex* and have a better understanding about the effect of oxidative condition on *S. spinosa* growth and spinosad and PSA production, the *rex* mutant (*S. spinosa* Lu106) was constructed. Cell growth, spinosad and PSA production, NADH/NAD^+^ levels, and gene expressions of *cytA* and *cytB* of *S. spinosa* Lu106 were studied. As shown in Figure 1A, the growth of *S. spinosa* Lu106 exhibited a growth defect relative to that of the wild type. Besides, the entry into stationary phase of *rex* mutant was delayed relative to that of the wild type (Figure 1A). The yield of spionsad and PSA in *rex* mutant was severely decreased (Figure 1C). The NADH/NAD^+^ levels in *rex* mutant were most stable during the whole fermentation process and maintained at a lower level (Figure 2). As shown in Figure 3, *cytA* and *cytB* were expressed from the beginning the fermentation. The expression of these two genes was very stable during the lag phage and exponential phase (Figure 3). At the stationary phase, the expression ratios increased (Figure 3). These results indicated that the expression of *cytAB* was regulated not only by *rex* but also some other genes. These results pointed out that cytochrome *bd* oxidase (*cytAB*) and many NADH dehydrogenases were continuously expressed in the *rex* mutant, which will consume many metabolites and NADH. As a result, cell growth and other compound synthesis, such as spinosad and PSA, were repressed.

### Enzyme activities analysis

**Figure 4:**
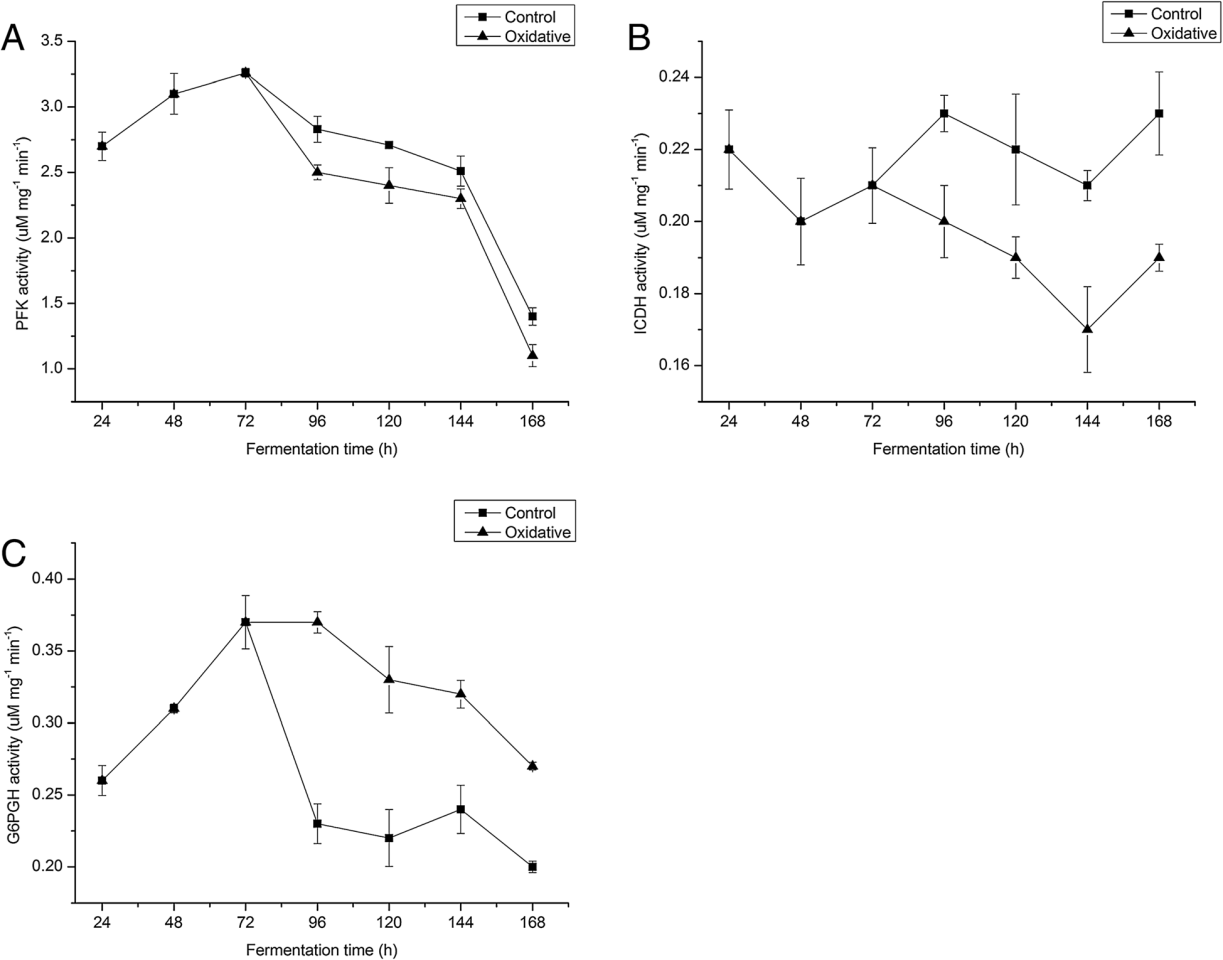
**Activities of PFK, ICDH, and G6PDH under control condition and oxidative condition of wild-type** ***S. spinosa*** **.** Activities of PFK **(A)**, ICDH **(B)**, and G6PDH **(C)** under control condition (square) and oxidative condition (triangle) of wild-type *S. spinosa*.

Further insights into the physiological consequences caused by oxidative condition were obtained by determining the activities of key redox-dependent enzymes (PFK, ICDH and G6PDH) in glycolysis, TCA cycle, and PPP. Although the activities of PFK in the stationary phage decreased with the time in both the control group and the oxidative condition, PFK activities decreased more sharply under oxidative condition than that in the control group in the whole stationary phase (Figure 4A). As shown in Figure 4B, the activities of ICDH in the control group (0.22 uM mg^−1^ min^−1^) was different from (P < 0.05) that in the oxidative group (0.2 uM mg^−1^ min^−1^) during the whole stationary phage. As shown in Figure 4C, G6PDH activities show opposite results to PFK and ICDH. The activities of G6PDH under oxidative condition were much higher than that in the control group (Figure 4C).

### Intracellular metabolites analysis

**Figure 5:**
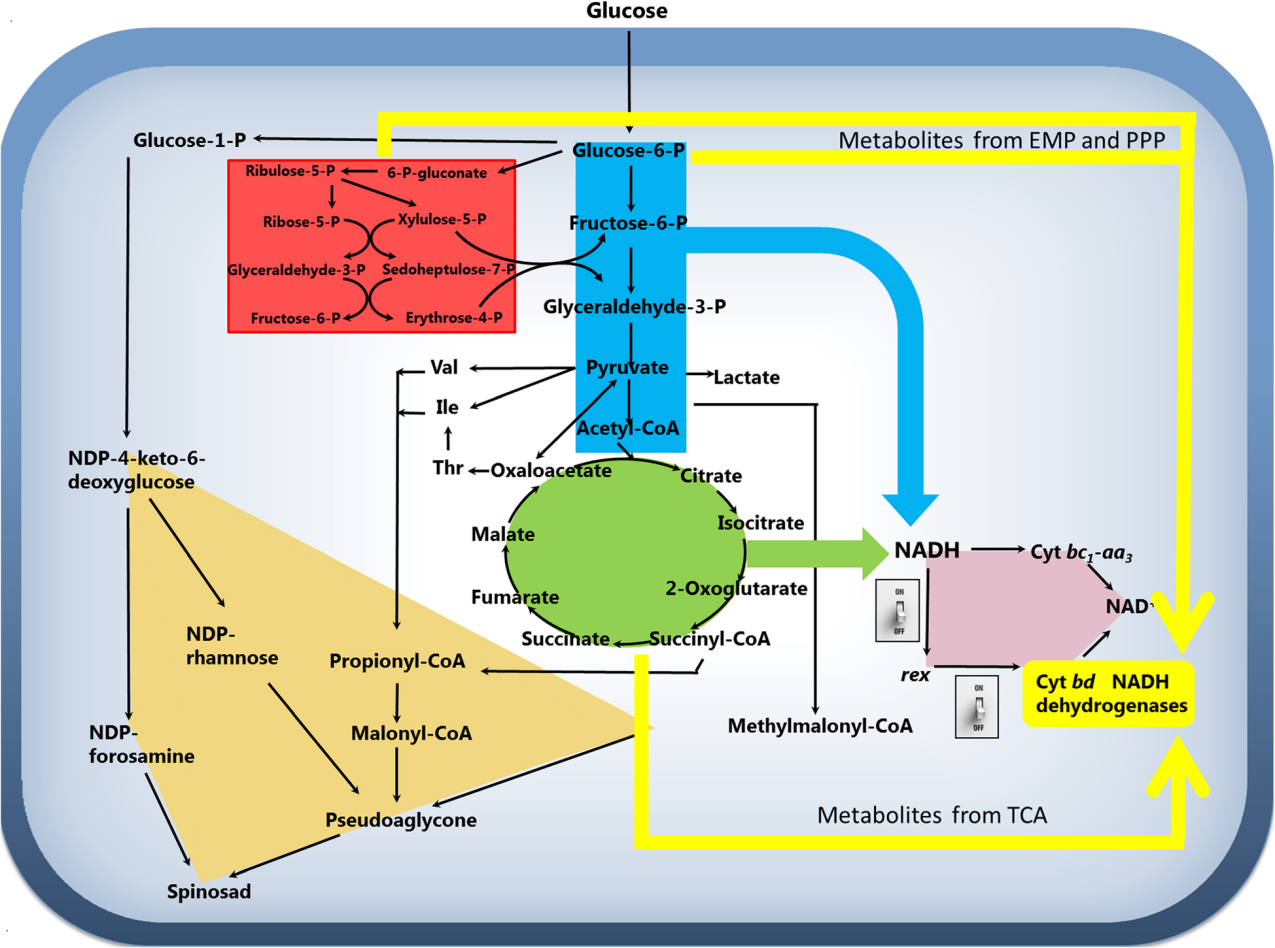
***Rex*** **regulation on the metabolism of** ***S. spinosa*** **.** EMP module was shown as blue background, TCA cycle, PPP was shown as red background; spinosad and PSA biosynthesis were shown as brown background; intermediates from EMP, PPP, and TCA flow to *rex*-controlled genes were shown in yellow line.

As we have shown, the oxidative condition can influence *S. spinosa* growth, spinosad and PSA production, *rex* DNA binding ability which determines the expression of many NADH dehydrogenases and cytochrome *bd* oxidases, and the key enzyme activities involved in glycolysis, TCA cycle and PPP. To obtain a detailed relationship between central carbon metabolism changes and spinosad synthesis, intracellular metabolites were analyzed by GC-MS and HPLC both in the control group and oxidative group (Additional file 2: Table S1). Metabolites involved in the central carbon metabolism and spinosad synthesis were determined (Table 1). As shown in Table 1, the concentrations of key metabolite 6-phophogluconate, involved in PPP were almost the same between the oxidative group and the control group during the whole stationary phase. In contrast, concentrations of key metabolites in glycolysis, citrate cycle, and spinosad synthesis were all higher under oxidative condition than that in the control. So, higher production of PSA and spinosad would be resulted from the higher concentrations of these central carbon metabolites and spinosad synthesis related metabolites. A whole metabolic explanation was illustrated in Figure 5.

**Table 1:** **the concentrations of key metabolites involved in glycolysis, citrate cycle, pentose phosphate pathway and spinosad synthesis under the control and oxidative condition**

**Metabolites**	**72 h**		**96 h**		**120 h**		**144 h**		**168 h**	
	**Control** ^**a**^	**Oxidative**	**Control**	**Oxidative**	**Control**	**Oxidative**	**Control**	**Oxidative**	**Control**	**Oxidative**
**Glycolysis**										
Fructose-6-P	1	1	1.13	1.62	0.94	1.35	1.26	0.75	0.67	0.93
glyceraldehyde 3-phosphate	1	1	0.97	1.54	1.00	2.09	0.94	1.21	0.96	0.53
Pyruvate	1	1	1.26	1.56	1.79	1.24	0.81	1.50	1.16	1.38
Acetyl-CoA	1	1	1.31	1.79	1.06	2.53	1.22	0.97	0.52	0.89
L-Lactate	1	1	2.32	0.35	1.39	ND	1.16	0.17	1.63	ND
**Pentose phosphate pathway**										
Glucose-6-P	1	1	1.74	6.20	2.16	7.22	1.92	7.16	1.31	4.97
6-phosphogluconate	1	1	0.73	0.81	0.44	0.53	0.25	0.21	ND	0.14
**Citrate cycle**										
Citrate	1	1	1.29	2.89	1.12	1.96	0.93	1.89	0.77	1.37
Oxaloacetate	1	1	0.59	1.28	0.41	1.05	0.37	0.92	0.46	0.79
Succinyl-CoA	1	1	1.62	3.42	1.73	4.11	1.07	3.21	0.93	3.07
**Spinosad synthesis related**										
Threonine	1	1	1.16	1.39	0.50	0.85	0.26	0.68	ND	0.42
Valine	1	1	1.14	2.69	1.69	3.99	1.92	3.51	0.25	0.73
Isoleucine	1	1	0.51	1.17	0.27	0.86	0.20	0.57	0.26	0.45
Propionyl-CoA	1	1	1.47	2.73	1.94	3.16	1.86	3.37	1.66	2.79
Malonyl-CoA	1	1	1.24	1.99	1.17	1.48	0.97	1.72	1.10	1.91
Methylmalonyl-CoA	1	1	1.05	1.50	1.03	1.44	1.21	1.90	0.88	1.16

^a^:The concentration at 72 h was the set as 1; ND: Under the lower limit of detection.

## Discussion

It has been found that under oxidative conditions, more flux flow through the synthesis of spinosad and cell growth, less flux flow through the synthesis of PSA and spinosad under reductive conditions. These results indicated that extracellular ORP can influence the metabolic flux. This is consistent with Christophe’s study which demonstrated that extracellular ORP can modify carbon and electron flow in *E. coli* [16]. In our study, DTT and H_2_O_2_ were used to modify the extracellular ORP. Because of the toxicity of high concentration of H_2_O_2_, we chose to add H_2_O_2_ every 12 h to create the oxidative condition. Because the addition of H_2_O_2_ can improve the yield of PSA and spinosad, further study about the response of *S. spinosa* was performed.

During the stationary phase, NADH/NAD^+^ ratios in the control group were higher than that in the oxidative group (Figure 2). In the control group, NADH/NAD^+^ ratios in the stationary phase were higher than that in the lag phase and exponential stage (Figure 2). However, NADH/NAD^+^ ratios in the stationary phase were more stable and almost the same as that in the lag phase and exponential stage under the oxidative condition. Studies have demonstrated that H_2_O_2_ is electron acceptor [17]. During the fermentation process, H_2_O_2_ accepted electrons from NADH directly or was degraded to H_2_O and O_2_. As a result, part of NADH was oxidized by H_2_O_2_ that resulted in the lower NADH/NAD^+^ ratios under oxidative condition. During the fermentation of *Actinomycetes*, high stirring speed damages the mycelium [18]. And the mycelium morphology of *Actinomycetes* plays an important role in polyketides production [19]. Our study found that electron acceptors can be provided without increasing stirring speed, which would damage the mycelium morphology of *Actinomycetes*.

*Rex* is a sensor of NADH/NAD^+^ in many Gram-positive bacteria, including *S. coelicolor* [11], *S. erythraea* [15], and *B. subtilits* [20]. By sensing cellular NADH/NAD^+^, *rex* regulates the transcription of many genes involved in central carbon metabolism, NADH reoxidation, such as cytochrome *bd* oxidase (*cytAB*) and NADH dehydrogenases to maintain cellular redox balance [11]. In the *rex* mutant *cytA* and *cytB* were expressed in the whole fermentation process, which indicated that the expression of *cytA* and *cytB* was influenced by *rex* in *S. spinosa*. We also found that the expression of *cytA* and *cytB* was also influenced by other regulation factors (Figure 3). Under oxidative condition, *cytA* and *cytB* were not expressed. This indicated that the DNA binding ability of *rex* was abolished. This result is consistent with NADH/NAD^+^ ratios in oxidative condition (Figure 2). It also indicates that many other NADH dehydrogenases, such as alcohol dehydrogenase and lactate dehydrogenase, were not expressed.

The activities of three key enzymes involved in EMP, TCA, and PPP were analyzed. Although studies have demonstrated that in vitro measured enzymatic activity do not obligatorily correlate with in vivo metabolic fluxes [21], enzyme activity can provide some information about metabolic changes. The lower activities of PFK and ICDH under oxidative condition than that in the control group indicated that PFK and ICDH were allosterically inhibited by higher metabolites concentrations in EMP and TCA under oxidative condition [22]. Metabolites measurement certified the enzyme activity result. Although metabolites involved in PPP under oxidative condition were higher than that in the control group, G6PDH activity under oxidative condition was still higher than that in the control group. G6PDH activity results indicated that PPP pathway was significantly up-regulated under oxidative condition.

The synthesis of spinosad and PSA requires many primary metabolites, such as acetyl-CoA, propinyl-CoA, NADPH, and succinyl-CoA, in the stationary phase of the fermentation [23,24]. As shown in Table 1, the concentrations of spinosad and PSA direct precursors, glucose-6-P, acetyl-CoA, propionyl-CoA, malonyl-CoA, and methylmalonyl-CoA in oxidative group were much higher than that in the control group. Besides, the concentrations of acetyl-CoA and propionyl-CoA related precursors, succinyl-CoA, threonine, valine, and isoleucine were also higher in oxidative group. Taken together we found that the concentrations of most of precursors related to spinosad and PSA synthesis was higher under oxidative condition than that in control condition (Table 1). The gene expression results indicated that the DNA binding ability of *rex* was abolished under oxidative condition. Because of the inhibition of *rex* regulation, many NADH dehydrogenases and inefficient terminal oxidases (cytochrome *bd*) were not expressed. So lots of metabolites were not waste to balance NADH/NAD^+^ metabolism under oxidative condition. The explanation of the whole process was illustrated in Figure 5.

## Conclusions

The regulative function of rex was inhibited by adding extracellular electron acceptor-H_2_O_2_ in the stationary phase. Under this condition, many NADH dehydrogenases which were used to balance NADH/NAD^+^ by converting useful metabolites to useless metabolites and inefficient terminal oxidases (cytochrome *bd*) were not expressed. So lots of metabolites were not wasted to balance. As a result, un-wasted metabolites related to spinosad and PSA synthesis resulted in a high prodution of spinosad and PSA under oxidative condition (Figure 5).

## Methods

### Strains, mutant construction and growth conditions

Plasmids and stains used in this study are listed in Table 2. *Escherichia. coli* DH5α and Top10 were used for plasmid construction and amplification. *E. coli* S17-1 was used as the door strain in biparental intergeneric conjugations. *Saccharopolyspora spinosa* ATCC 49460 was used as the parent strain.

**Table 2:** **The strains and plasmids used in this study**

**Strain or plasmids**	**Description**	**Source or reference**
**Strains**		
*E. coli* DH5α	Host for general cloning	TransGen Biotech
*E. coli* TOP10	Host for general cloning	TransGen Biotech
*E. coli* S17-1	Donor stain for conjugation between *E. coli* and S. spinosa	[25]
*S. spinosa* ATCC 49460	Wild strain	[26]
*S. spinosa* Lu106	*S. spinosa* ATCC 4946 with pLu106	This study
**Plasmids**		
POJ260	*E. coli* – *Streptomcyes* shuttle vector; *apr oriT rep* ^*PUC*^ *lacZ*	[27]
pLu106	pOJ260 with truncated *Rex*	This study

Oligonucleotide primers used in this study are listed in Table 3. To construct *rex* mutant *S. spinosa*, first, part of *rex* (604 bp) fragment was amplified from genomic DNA of *S. spinosa* using primer pairs of rex-F-HindIII, rex-R-XbaI. Then the 604 bp fragment was digested by HindIII (Fermentas) and XbaI (Fermentas) and ligated to pOJ260 obtaining pLu106. pLu106 was introduced into *S. spinosa* ATCC 49460 by conjugation from *E. coli* S17-1 and homologous recombination into the chromosome as described previously [28]. The plasmid was inserted into the middle *rex* of *S. spinosa* ATCC 49460 to create *S. spinosa* △*rex* (Lu106). *S. spinosa* △*rex* was confirmed by PCR amplification with primers Con-F and Con-R.

**Table 3:** **Sequences of oligonucleotide primers used in this study**

**Primers**	**Sequence 5’ → 3’**
rex-F-HindIII	CT**AAGCTT**TGTCCGCACTCGCCGAC
rex-R-Xbal	CT**TCTAGA**ATCCACATCGGATCGATCGG
cydA-F	TATCGCACCGGCAAGCAG
cydA-R-	GAACTCCTGCACGATGCC
cydB-F	GATCTGCCCACCTTCTGG
cydB-R-	CATGCCGACGCCGAAGTC
Con-F	CCGTGATTTTGTAGCCCTGG
Con-R	GGCCTACTTCACCTATCCTGC
16S rRNA-F	CCTACGAGCTCTTTACGCCC
16S rRNA-R	AGAAGCACCGGCTAACTACG
rbL13-F	GGCGTAGACCTTGAGCTTC
rbL13-R	GCTCGAAAAGGCGATCAAG

*E. coli* strains were grown at 37°C in Luria-Bertani medium. Apramycin was used as a selection agent at 100 ug/ml for *E. coli* and at 50 ug/ml for *S. spinosa. S. spinosa* were cultured as described [8]. First, *S. spinosa* was cultured for 3 days in seed medium (g/L) which was composed by Trypticase soy broth, 30; yeast extract, 3; MgSO_4_ · 7H_2_O, 2; glucose, 10; and maltose, 4, pH 7.2. Then 3 mL of seed medium were injected into 30 mL fermentation medium (g/L) which was composed by glucose, 68; cottonseed flour, 22; peptone C, 25; corn seed liquor, 14.5; methyl oleate, 40; and CaCO_3_, 5, pH 7.2. The fermentation medium was optimized by response surface methods [10].

### Determination of spinosad and *S. spinosa* growth

Spinosad in fermentation broth was extracted and determined by HPLC as described [10]. Dry cell weight (DCW) was determined as described [29]. Glucose was measured by using the dinitrosalicylic acid (DNS) method [30]. The experiments were repeated three times.

### NADH and NAD^+^ extraction and determination

NADH and NAD^+^ were extracted according to a previous described method with some modifications [31]. 5 mL cell cultures were collected, chilled on ice immediately, and centrifuged at 12000 g, 4°C for 10 min. Then cell pellets were immediately ground to powder in a porcelain mortar, which was pre-cooled to −80°C, under liquid nitrogen for 5 min. After that, NADH was extracted by the addition of 300 uL 0.2 mol/L NaOH. NAD^+^ was extracted by the addition of 300 uL 0.2 mol/L HCl. Then the samples were heated at 50°C for 10 min and neutralized using NaOH or HCl. After neutralization, the samples were centrifuged at 12000 g, 4°C for 10 min. The supernatant was collected and stored at −80°C until used. NADH and NAD^+^ in the supernatant were determined using NAD/NADH quantitation kit (Comin), according to manufacturer’s instructions. The kit is based on an enzymatic cycling assay method.

### Enzyme activity assays

20 mL cell cultures were collected, chilled on ice immediately, and centrifuged at 3000 g, 4°C for 10 min. Cell pellets were suspended in 2 mL Tris–HCl buffer (100 mM, pH 7.2) and disrupted by sonication on ice for 5 min (pulse intensity 40%, pulse on for 10 s and off for 50s). After centrifugation (12000 g, 4°C for 30 min), the supernatant was used for enzyme assay. 6-phosphofructokinase (PFK) activity was determined as described [31]. Isocitrate dehydrogenase (ICDH) activity was determined by measuring the production of NADH [32]. Glucose-6-phophate dehydrogenase (G6PDH) activity was carried out by measuring the formation of NADPH as described previously [33].

### RNA extraction, cDNA synthesis, and real-time qPCR analysis

RNA extraction, cDNA synthesis, and real-time qPCR analysis of *S. spinosa* were performed as described previously [34]. 16S rRNA and rbL13 were used to normalize the qPCR data. The primers used in qPCR are listed in Table 3.

### Intracellular metabolites using GC-MS

4 mL cell cultures were mixed with 6 mL cold methanol (−40°C) to arrest metabolism instantaneously. Then, samples were centrifugated at 3000 g for 3 min. Cell pellets were collected and immediately ground to powder in a porcelain mortar, which was pre-cooled to −80°C, under liquid nitrogen for 5 min. Then 100 mg cell powder was mixed thoroughly with 1 ml −40°C 50% methanol (methanol/water, 1:1). The samples were centrifugated at 10000 g for 10 min. The supernatants were collected. Then 10 uL internal standard solution succinic d_4_ acid (Sigma, 0.1 mg/ml) was added into the 100 uL extract supernatants before lyophilization. After lyophilization, the derivatization and measurement by GC-MS of these samples were carried out according to a previous method [35]. Four biological replicates were performed for each sample. The identification and quantification of GC-MS peaks were performed using Agilent software (G1701DA MSD ChemStation ver. D.00.00.38).

### Metabolites involved in Spinosad synthesis determination

Short chain coenzyme A (CoA) in *S. spinosa* was extracted as described [8]. Acetly-CoA, malonayl-CoA, methylmalonyl-CoA, succinyl-CoA, and propionyl-CoA were measured by HPLC as described [36]. Pseudoaglycones (PSA), the intermediates of spinosad, was determined by HPLC as described [37].

## Additional files

Additional file 1: Figure S1. Multiple alignments of proteins from Rex family. Additional file 2: Table S1. The intracellular metabolites involved in Carbohydrate metabolism, Energy metabolism, Lipid metabolism, Amino acid metabolism, Nucleotide metabolism and spinosad pathway were analyzed by GC-MS and HPLC both in the control group and oxidative condition.
