# Supplementary figures and images for: Suitable extracellular oxidoreduction potential inhibit rex regulation and effect central carbon and energy metabolism in Saccharopolyspora spinosa
Source: Microb Cell Fact. 2014 Aug 27;13:98. doi: 10.1186/s12934-014-0098-z (PMC4172946; doi:10.1186/s12934-014-0098-z)

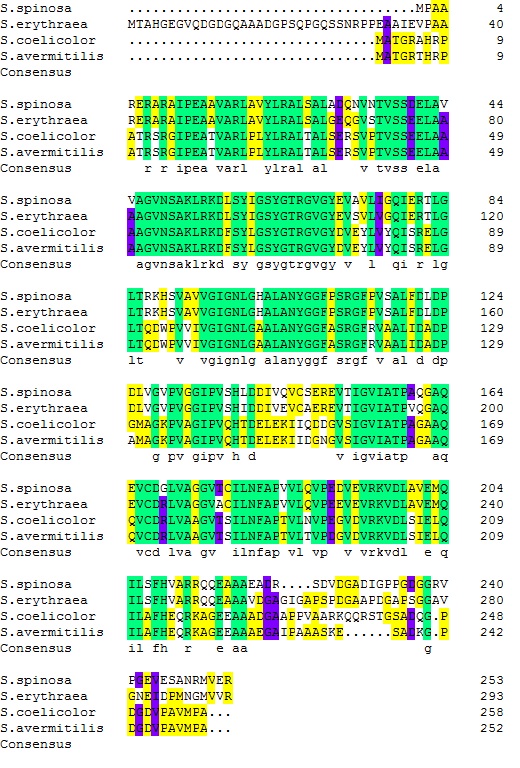

Supplement: Additional file 1: Figure S1. — Multiple alignments of proteins from Rex family. [file 12934_2014_98_MOESM1_ESM.jpeg]
